# Cardiovascular risk and use of conicity index in patients submitted to autologous hematopoietic stem cell transplantation

**DOI:** 10.1590/S1679-45082018AO4253

**Published:** 2018-05-29

**Authors:** Suelyne Rodrigues de Morais, Ana Carolina Cavalcante Viana, Ana Filomena Camacho Santos Daltro, Priscila da Silva Mendonça

**Affiliations:** 1 Hospital Universitário Walter Cantídio, Universidade Federal do Ceará, Fortaleza, CE, Brazil

**Keywords:** Hematopoietic stem cell transplantation, Transplantation, autologous, Obesity, abdominal, Cardiovascular diseases, Body mass index, Waist circumference, Transplante de células-tronco hematopoiéticas, Transplante autólogo, Obesidade abdominal, Doenças cardiovasculares, Índice de massa corporal, Circunferência da cintura

## Abstract

**Objective:**

To analyze the prevalence of overweight and the use of conicity index for cardiovascular risk assessment in individuals submitted to autologous hematopoietic stem cell transplantation.

**Methods:**

The sample comprised 91 patients of both sexes, who underwent autologous hematopoietic stem cell transplantation from September 2008 to December 2013, aged 18 years or over. To determine the nutritional profile, we collected anthropometric data on weight, height, waist circumference upon hospital admission. The body mass index and the conicity index were calculated.

**Results:**

A total of 91 patients diagnosed with multiple myeloma, Hodgkin's lymphoma, non-Hodgkin's lymphoma and other conditions were evaluated. The mean age was 43.5 (14.2) years, 63.7% were male. We verified that according to the body mass index, 63.7% were overweight and, according to waist circumference, 74.7% had a higher risk for cardiovascular diseases. According to the calculation of the conicity index, 92.3% of patients presented increased cardiovascular risk. Patients with multiple myeloma had a higher conicity index when compared to other patients (p<0.01).

**Conclusion:**

This study revealed a high prevalence of overweight and cardiovascular risk. It should be noted that the conicity index was a good method to evaluate cardiovascular risk and that new studies using this index should be performed.

## RESUMO

**Objetivo:**

Analisar a prevalência de excesso de peso e a utilização do índice de conicidade para a avaliação do risco cardiovascular em indivíduos submetidos ao transplante autólogo de células-tronco hematopoiéticas.

**Métodos:**

A amostra foi composta por 91 pacientes, de ambos os sexos, submetidos ao transplante autólogo de células-tronco hematopoiéticas, de setembro de 2008 a dezembro de 2013, com idade igual ou superior a 18 anos. Para traçar o perfil nutricional, foram coletados dados antropométricos, como peso, altura e circunferência da cintura, na admissão hospitalar. Calcularam-se o índice de massa corporal e o índice de conicidade.

**Resultados:**

Foram avaliados 91 pacientes, diagnosticados com mieloma múltiplo, linfoma de Hodgkin, linfoma não Hodgkin e outras patologias. A média de idade foi de 43,5 (14,2) anos, e 63,7% eram do sexo masculino. Verificou-se que, de acordo com o índice de massa corporal, 63,7% apresentavam excesso de peso e, segundo a circunferência da cintura, 74,7% possuíam maior risco para doenças cardiovasculares. De acordo com o cálculo do índice de conicidade, constatou-se que 92,3% dos pacientes apresentavam risco cardiovascular aumentado. Os pacientes com mieloma múltiplo apresentaram índice de conicidade mais elevado, se comparado aos outros pacientes (p<0,01).

**Conclusão:**

Este estudo revelou alta prevalência de excesso de peso e de risco cardiovascular. Salienta-se que o índice de conicidade se mostrou um bom método para avaliação do risco cardiovascular e que novos estudos utilizando este índice devem ser realizados.

## INTRODUCTION

Some parameters, such as weight gain and increased waist circumference (WC), are associated with a greater risk of developing several types of cancer. The risk of developing cancer can grow from 9 to 56% for every 5kg/m^2^ raise in the body mass index (BMI).^(^ ^1^ ^)^ Further, obesity is associated with increased cardiovascular morbidity and mortality, and in large part is a consequence of vascular abnormalities, resulting in increased risk of cardiac diseases and stroke.^(^ ^2^ ^)^

Patients submitted to hematopoietic stem cells transplantation (HSCT) present an increased risk of death by cardiovascular disease in comparison to healthy individuals.^(^ ^3^ ^)^ Currently, it is known that patients who survive HSCT may be at a higher risk of prematurely developing cardiovascular diseases (CVD) and metabolic syndrome.^(^ ^4^ ^)^

Excess weight is considered a risk factor for acute infections, increased complications, and mortality in the post-transplant period.^(^ ^5^ ^)^ Hence, patients should be offered early nutritional management, aiming to reduce metabolic complications, to maintain or improve nutritional statu, and provide adequate amount of nutrients and calories for hematopoietic recovery.^(^ ^6^ ^)^

Several anthropometric measurements may be used in the nutritional assessment of these patients.^(^ ^7^ ^)^ To evaluate total body obesity, the most frequently used indicator is the BMI.^(^ ^8^ ^)^ It is known, however, that this index does not evaluate body fat distribution, but rather the body as a whole (fat tissue and muscle tissue).^(^ ^9^ ^)^

Waist circumference WC is another widely used measurement of obesity, cited in literature as a good indicator of abdominal obesity, easier to measure and highly associated with CVD.^(^ ^10^ ^)^

At the beginning of the 1990s, the conicity index (C index) was proposed, which assesses obesity and distribution of fat tissue, considering that central obesity, more than total body obesity, is associated with increased diagnosis of CVD.^(^ ^11^ ^)^

The C index is a relatively new index, although it has already been used to evaluate abdominal adiposity in patients submitted to solid organ transplantation,^(^ ^12^ ^)^ but no study has analyzed the use of the C index in patients submitted to HSCT.

Due to the importance of associating more than one anthropometric marker in determining cardiovascular risk^(^ ^13^ ^)^ and bearing in mind the increased risk of CVD development in individuals submitted to HSCT,^(^ ^3^ ^,^ ^4^ ^)^ it is important to analyze the prevalence of excess weight and the use of C index in individuals submitted to autologous HSCT.

## OBJECTIVE

To analyze the prevalence of excess weight and the use of the conicity index to assess cardiovascular risk in individuals submitted to autologous hematopoietic stem cell transplant.

## METHODS

This is a quantitative cross-sectional investigation using secondary data from medical records, carried out at a university hospital in Fortaleza (CE), Brazil. It was approved by the Research Ethics Committee, under opinion 1.603.999, CAAE: 54190616.4.0000.5045.

The sample was made up of 91 patients of both sexes, submitted to autologous HSCT during the period from September 2008 to December 2013, which corresponds to the first five years of HSCT at the organization. Included were patients aged 18 years or more. Two individuals under the age of 18 years were excluded along with 16 others whose medical records were not found, with a total loss of 18 individuals.

Data collection was made by a single researcher using a pre-established form, with sociodemographic data, such as sex and age, date of HSCT, clinical diagnosis, and anthropometric data (weight, height, and WC upon hospital admission).

With weight and height data, the BMI was calculated consisting of the weight in kilograms divided by the square of the height, in meters. Cutoff points for the adult population were determined by the World Health Organization (WHO);^(^ ^14^ ^)^ namely,<18.5kg/m^2^ indicates malnutrition; between 18.5 and 24.9kg/m^2^, well-nourished; and ≥25kg/m^2^, excess weight. For the elderly population, the criteria proposed by Lipschitz were used:^(^ ^15^ ^)^ <22kg/m^2^ for low weight; between 22 and 27kg/m^2^ for well-nourished; and >27kg/m^2^ for excess weight. The classification as to WC followed the WHO recommendations^(^ ^8^ ^)^ (Table 1).

**Table 1:** Waist circumference values considered of risk for obesity-associated diseases

Sex	Low risk (cm)	High risk (cm)	Very high risk (cm)
Female	<80	≥80	≥88
Male	<94	≥94	≥102

Source: World Health Organization (WHO).

Obesity: preventing and managing the global epidemic. Report of a WHO Consultation. Geneva; WHO: 2000. [WHO Technical Report Series 894]^(^ ^8^ ^)^.

For the calculation of the C index, the measurements of weight, height, and WC were used, according to Valdez,^(^ ^11^ ^)^ by means of the following mathematical equation:

$$C\;\mathrm{index}=\frac{\mathrm{Waist}\;\mathrm{circumferernece}\;(m)}{0.109\sqrt{\frac{\mathrm{Body}\;\mathrm{weight}\;(\mathrm{kg})}{\mathrm{Height}\;(m)}}}$$

For cardiovascular risk calculation, the values proposed by Pitanga et al. were used, that is, ≥1.18 for women and ≥1.25 for men.^(^ ^16^ ^)^

Data were organized in tables and graphs. Statistical analysis was conducted by means of the Statistical Package for the Social Science (SPSS) software, version 22.0. The Kolmogorov-Smirnov test was used for the analysis of normality, and the Levene test, to verify homogeneity. We used Spearman's test for the correlations, and the χ^2^ test to verify associations. The variance analysis test (ANOVA) was used to check differences among the means, and for the post-test, we used Tukey. The significance level was set at 0.05 for all tests performed.

## RESULTS

The study was composed of 91 patients; of these, 63.7% were male. Mean age was 43.5 (14.2) years, range of 19 to 69 years. As to the baseline diagnosis of patients, the greatest prevalence was multiple myeloma. The mean age of patients differed among those diagnosed with multiple myeloma, Hodgkin's lymphoma, and non-Hodgkin's lymphoma, and the highest mean age was found in multiple myeloma patients (Table 2).

**Table 2:** Diagnosis of patients submitted to autologous hematopoietic stem cell transplantation

Diagnosis	n (%)	Mean age (SD)	p value
Multiple myeloma	49 (53.8)	53.8 (8.1)ᵃ	<0.001*
Hodgkin´s lymphoma	24 (26.4)	27.8 (5.7)^b^	
Non-Hodgkin´s lymphoma	15 (16.5)	37.8 (11.1)^c^	
Others†	3 (3.3)	29.0 (13.1)^bcd^	
Total	91 (100.0)	-	

ANOVA. Similar letters indicate there is no significant difference, and different letters indicate there is difference among the groups, according to Tukey test.

* p value considered significant when <0.05;

† seminoma, plasma cell leukemia and mediastinal embryonal carcinoma. SD: standard deviation.

According to the anthropometric assessment, the mean BMI of individuals was 27.28 (±4.47) kg/m^2^; of WC it was 96.83 (±11.27) cm; and of C index, it was 1.34 (±0.79). The mean BMI and WC did not differ among the several diagnoses. The mean C index was significantly higher among the individuals with multiple myeloma when compared to Hodgkin's lymphoma patients (Table 3).

**Table 3:** Mean body mass index, waist circumference and conicity index according to diagnosis in patients submitted to autologous hematopoietic stem cell transplantation

Variable	Diagnosis				p value
	Multiple myeloma Mean age (SD)	Hodgkin's lymphoma Mean (SD)	Non-Hodgkin's lymphoma Mean (SD)	Outros* Média (DP)	
BMI (kg/m^2^)	27.90 (3.08)	27.49 (6.63)	24.90 (3.88)	27.27 (2.47)	0.15
WC (cm)	96.8 (9.09)	95.31 (15.95)	92.93 (8.76)	95.67 (4.80)	0.28
C index	1.36 (0.07)ᵃ	1.29 (0.08)^b^	1.33 (0.05)^ab^	1.31 (0.07)^ab^	<0.01†

ANOVA. Similar letters indicate there is no significant difference, and different letters indicate there is difference among the groups, according to Tukey test.

* seminoma, plasma cell leukemia and mediastinal embryonal carcinoma.

† p value considered significant when <0.05.

SD: standard deviation; BMI: body mass index; WC: waist circumference; C index: conicity index.

According to the C index calculation, it was determined that 92.3% of patients presented with an increased cardiovascular risk (Table 4). There was also a significant correlation between the C index and patients’ age (r=0.418; p<0.001). No significant associations were found among sex and BMI classifications, WC, and C index.

**Table 4:** Characterization of nutritional status and cardiovascular risk of patients submitted to autologous hematopoietic stem cell transplantation

Variable		Sex		Total n (%)	p value
		Female n (%)	Male n (%)		
BMI					0.45
	Malnourished	0 (0.0)	1 (1.7)	1 (1.1)	
	Well-nourished	14 (42.4)	18 (31.0)	32 (35.2)	
	Excess weight	19 (57.6)	39 (67.3)	58 (63.7)	
WC					0.64
	Low risk	4 (12.1)	19 (32.8)	23 (25.3)	
	High risk	4 (12.1)	17 (29.3)	21 (23.1)	
	Very high risk	25 (75.8)	22 (37.9)	47 (51.6)	
C index					0.45
	With risk	31 (93.9)	53 (91.4)	84 (92.3)	
	No risk	2 (6.1)	5 (8.6)	7 (7.7)	

Pearson χ^2^. BMI: body mass index; WC: waist circumference; C index: conicity index.

## DISCUSSION

Most of the participants were male, and the most prevalent condition was multiple myeloma, as identified by Pereira et al.,^(^ ^17^ ^)^ who evaluated the nutritional status of patients submitted to autologous HSCT.

In this study, a high prevalence of excess weight was detected in patients submitted to HSCT (63.7%), corroborating growing evidence of an association between excess weight and diagnosis of hematological cancer.^(^ ^1^ ^,^ ^17^ ^)^

Hadjibabaie et al.,^(^ ^18^ ^)^ assessed the nutritional status of patients submitted to autologous or allogenic HSCT, and found a prevalence of excess weight in 28% of patients, that is, lower than that found in this study (63.7%). However, Vogl et al.,^(^ ^19^ ^)^ demonstrated a high prevalence of excess weight in multiple myeloma patients (73.1%), based on BMI. Nevertheless, it is important to consider that BMI does not distinguish excess body fat from lean mass, and is more reliable when associated with other anthropometric measurements.^(^ ^20^ ^)^

Over the last years, the diagnosis of obesity has evolved, showing that the use of BMI may not precisely identify the cardiovascular risk related to obesity. Individuals with a “well-nourished” BMI and a high content of body fat present with a high risk of inflammation, metabolic deregulation, and increased mortality.^(^ ^21^ ^)^

Waist circumference also showed high prevalence of excess abdominal fat tissue in these patients. Additionally, this anthropometric measurement is a simple and effective method to check total and intraabdominal fat.^(^ ^22^ ^)^ Liu *et al.*,^(^ ^23^ ^)^ investigated the nutritional status of Chinese patients before and after HSCT, and in the pre-transplant period, a mean WC of 84.9cm was measured and presented low risk for obesity-related diseases. Whereas in the study by Bhansali et al.,^(^ ^24^ ^)^ which evaluated patients submitted to autologous HSCT in India, an increased mean WC (103cm) was noted, and the value was higher to that found in the present study (96.83cm).

Central obesity has proven more effective for assessing cardiovascular risk when compared to total body obesity.^(^ ^25^ ^)^ We further point out that the accumulation of fatty tissue in the abdominal region has been mentioned as a risk factor for CVD and for increased mortality, even in the absence of excess weight.^(^ ^26^ ^)^

Still, as to the cardiovascular risk, the values were higher according to the C index (92.3%), showing that it is a good anthropometric method to assess this risk despite being less practical than WC, due to the need of calculations. The C index considers that individuals that accumulate fat around the central regional of the trunk have a conical body shape, while people with a smaller quantity of fat in the central region would have a cylindrical appearance.^(^ ^27^ ^)^ Pitanga et al.,^(^ ^28^ ^)^ observed that the C index was the best predictor for cardiovascular complications, comparing it with other anthropometric measurements, such as BMI and WC. Moreover, this parameter can be used to determine abdominal obesity in clinical practice, when associated with other measurements.

In addition to its importance to evaluate central obesity, the C index has been negatively associated with concentrations of cardiovascular protection biomarkers, such as HDL cholesterol.^(^ ^29^ ^)^ No studies were found that used the C index in anthropometric evaluation of individuals submitted to HSCT, or in analysis of hematologic and oncological diseases; the present study stands out for having unpublished data.

In this regard, based on the results obtained, verifying cardiovascular risk in patients who are candidates to HSCT is of utmost importance, considering these patients are submitted to direct and indirect cardiovascular lesions, and CVD is a frequent and devastating complication, leading to low quality of life and increased morbidity and mortality.^(^ ^30^ ^)^

Aware of the fact that all candidates for autologous HSCT are considered at high nutritional risk, nutritional therapy is a great challenge. According to the national consensus of oncologic nutrition, patients should receive adequate and effective nutritional support during all phases of the transplant, besides periodic follow-up of the common comorbidities and complications in HSCT.^(^ ^6^ ^)^

A few limitations of this study are related to data collection, performed in manually written medical records, thus hindering acquisition of all pieces of information due to loss of medical records.

The data presented contributed towards the adoption of nutritional evaluation and approach in all stages of HSCT. Further studies using the C index shall be conducted to verify the interpretation of this instrument in the nutritional assessment and follow-up of cardiovascular risk.

## CONCLUSION

This study showed a high prevalence of excess weight, based on the body mass index, in patients submitted to autologous hematopoietic stem cell transplantation. Increased cardiovascular risk was detected in a large part of the patients, based on both waist circumference and the C index measurements. We emphasize that the C index proved to be a good method to assess cardiovascular risk, and can be used together with waist circumference and body mass index.
